# Royal Chest Hospital Dinner

**Published:** 1914-05-16

**Authors:** 


### Royal Chest Hospital Dinner.

### IMPORTANT ANNOUNCEMENT.

The Royal Chest Hospital, in the City Road, is trying to take full advantage of the new order of things thoracic consequent upon the intervention of the State in the treatment of tuberculosis. As we have already recorded, a tuberculosis dispensary has been doing good work for some eighteen months in the new out-patient department, and now the council has decided to embark on a scheme for the badly needed rebuilding of the hospital itself. An appeal for the sum of £16,000 is shortly to be issued, but towards this a very respectable proportion has already been promised. This announcement, as well as some interesting statements about the new medical scheme, was made by Dr. Barty King at the medical staff dinner held on Friday, last week, at the Savoy Hotel.

This dinner served at one and the same time to mark the centenary of the hospital and the retirement of the senior physician, Dr. W. H. White, after thirty-four years' active service in the interests of the institution. The dinner was followed by an unusually entertaining set of speeches led by Sir R. Douglas Powell. A *résumé* of the history of the Chest Hospital was given by Dr. Arthur Davies, who outlined its remarkable career since 1814. Many distinguished guests from other chest hospitals were present, on behalf of whom Sir St. Clair Thomson and Dr. Mitchell Bruce replied to the toast proposed by Sir Alfred Pearce Gould. Dr. Samuel West proposed the health of the Chairman, Dr. White, who gave in return some of his reminiscences. In reply to the toast of the medical school, proposed by Professor Sims Woodhead, the Dean, Dr. Barty King, told of the successes of the past and the hopes of the future in connection with post-graduate work, and the effect of the Insurance Act on the medical practitioner. Among the recent innovations in the City Road must be mentioned the appointments just about to be made of a director of the clinical, pathology, and research laboratory and a medical registrar, and also the installing of an electrocardiograph.

